# Deleterious impact of trivial to severe interstitial pneumonia and emphysema on mortality and acute exacerbation of interstitial pneumonia in patients with lung cancer: a retrospective cohort study

**DOI:** 10.1186/s12890-024-03105-7

**Published:** 2024-06-22

**Authors:** Yutaka Tomishima, Atsushi Kitamura, Ryosuke Imai, Sachiko Ohde

**Affiliations:** 1 Department of Pulmonary Medicine, Thoracic Center, St. Luke’s International Hospital, St. Luke’s International University, 9-1 Akashi-cho, Chuo-ku, Tokyo, 104-8560 Japan; 2 Graduate School of Public Health, St. Luke’s International University, 9-1 Akashi-cho, Chuo- ku, Tokyo, 104-8560 Japan

**Keywords:** Acute exacerbation of interstitial pneumonia, Combined pulmonary fibrosis and emphysema, Interstitial lung abnormalities, Lung neoplasms, Pulmonary emphysema, Interstitial pneumonia

## Abstract

**Background:**

Interstitial pneumonia and emphysema may complicate patients with lung cancer. However, clinical significance of trivial and mild pulmonary abnormalities remains unclear. In this study, we aimed to investigate whether trivial and mild interstitial pneumonia and emphysema, in addition to their advanced forms, impact the prognosis and lead to acute exacerbation of interstitial pneumonia (AEIP) in patients with lung cancer.

**Methods:**

This retrospective cohort study was conducted at a tertiary hospital and included patients with lung cancer. Computed tomography images were evaluated using the interstitial lung abnormality (ILA) score for interstitial pneumonia, which included no ILA, equivocal ILA, ILA, interstitial lung disease (ILD), and the Goddard score for emphysema. Cox analyses were performed using the ILA and Goddard scores as the main explanatory variables, adjusting for multiple covariates.

**Results:**

Among 1,507 patients with lung cancer, 1,033 had no ILA, 160 had equivocal ILA, 174 had ILA, and 140 had ILD. In total, 474 patients (31.5%) exhibited interstitial pneumonia and 638 (42.3%) showed emphysema. The log-rank trend test showed that survival probability was significantly better in patients with no ILA, followed by those with equivocal ILA, ILA, and ILD (*P* < 0.001). After adjustment, the ILA and Goddard scores remained significant variables for increased hazard ratios (HR) for mortality: no ILA (HR, 1.00: reference), equivocal ILA (HR, 1.31; 95% confidence interval [CI], 1.18–1.46; *P* < 0.001), ILA (HR, 1.71; 95% CI, 1.39–2.12; *P* < 0.001), ILD (HR, 2.24; 95% CI, 1.63–3.09; *P* < 0.001), and Goddard score (HR, 1.03; 95% CI, 1.01–1.06; *P* < 0.010). Moreover, both scores were associated with increased cause-specific HRs for AEIP.

**Conclusion:**

Our results revealed that approximately one-third of patients with lung cancer had interstitial pneumonia when incorporating trivial and mild cases. Because interstitial pneumonia and emphysema, ranging from trivial to severe, significantly impact mortality and AEIP in patients with lung cancer, we should identify even trivial and mild cases of these pulmonary abnormalities among patients with lung cancer in addition to the advanced ones.

## Background

Lung cancer is the leading cause of death among patients with cancer in several countries [1, 2]. Idiopathic pulmonary fibrosis (IPF) can complicate some cases of lung cancer, leading to poorer prognosis [3–5]. Moreover, any treatment for lung cancer could result in fatal acute exacerbation of IPF [6, 7]. IPF is the most common type of interstitial pneumonia [8]; however, the impact of overall interstitial pneumonia on the prognosis of lung cancer remains unclear [9]. Furthermore, the impact of trivial and mild extent of interstitial pneumonia on both lung cancer-related mortality and acute exacerbation of interstitial pneumonia remains unclear.

Interstitial lung abnormality (ILA) is defined solely based on computed tomography (CT) findings [10]. ILA includes various forms of interstitial pneumonia and early subclinical interstitial pneumonia [10]; thus, evaluation of ILA can help understand the effects of most interstitial pneumonia in patients with lung cancer. Previous studies on ILA used the ILA score [11, 12], which classifies the findings of chest CT into no ILA, equivocal ILA, ILA, and interstitial lung disease (ILD). Few studies have concluded that ILA is associated with poorer survival in patients with lung cancer [11–13]. However, to our knowledge, the adverse impact of equivocal ILA is not recognized in such patients.

Interstitial pneumonia is often associated with emphysema [14], and some studies have shown that emphysema is associated with a poorer prognosis in patients with lung cancer [15–17]. The concept of combined pulmonary fibrosis and emphysema (CPFE) [18, 19] allows concurrent evaluation of the effects of these two conditions in patients with lung cancer. Nevertheless, the interaction between interstitial pneumonia and emphysema has not been evaluated in conventional studies of CPFE [20–25]. Moreover, the extent of interstitial pneumonia and emphysema was not considered in studies of CPFE in patients with lung cancer.

In this study, we aimed to evaluate the prevalence of interstitial pneumonia and emphysema in patients with lung cancer using the ILA and emphysema scores. Furthermore, we aimed to test the hypothesis that equivocal ILA, ILA, and ILD have a progressively deleterious impact on all-cause mortality and acute exacerbation of interstitial pneumonia (AEIP) in patients with lung cancer. Additionally, we evaluated the comprehensive impact of interstitial pneumonia and emphysema on these outcomes using the ILA and emphysema scores, as well as the interaction between interstitial pneumonia and emphysema.

## Methods

### Study population

This retrospective cohort study was conducted at a tertiary hospital with 520 beds in Japan. The study population comprised all patients who were pathologically or cytologically diagnosed with non-small cell lung cancer and small cell lung cancer in all clinical stages from January 1, 2008, to December 31, 2020. We excluded patients whose chest CT data were unavailable before treatment.

### Evaluation of chest CT

Chest CT performed around the time of diagnosis of lung cancer was evaluated for the extent of interstitial pneumonia, extent of emphysema, and clinical stage of lung cancer. When available, high-resolution CT scans were assessed. Follow-up CT performed after the initiation of treatment for lung cancer was only used to ascertain the development of AEIP or evaluate possible gravity-dependent atelectasis that was difficult to distinguish from equivocal ILA in previously assessed CT images.

The extent of interstitial pneumonia was evaluated using the ILA score, which was obtained using the sequential reading method described by Washoko et al. [26]. Three independent pulmonologists evaluated each CT image and assigned one of four scores independent of clinical data: 0, no ILA; 1, equivocal ILA; 2, ILA; and 3, ILD. The ILA score was based on findings such as honeycombing, traction bronchiectasis, nondependent ground-glass opacity, nonemphysematous cysts, and reticular abnormalities. Abnormalities such as diffuse centrilobular nodularity, pleuro-pulmonary fibroelastosis, gravity-dependent atelectasis, and abnormalities associated with lung cancer, such as lymphangitic carcinomatosis, were excluded. Each lung was divided into three zones according to the levels of the inferior aortic arch and right inferior pulmonary vein [10], and the condition was classified as equivocal ILA, ILA, and ILD when the abnormalities mentioned above involved < 5%, > 5%, and > 20% of any lung zone, respectively. According to a previous study on ILA, if ILA involves more than three lung zones or presents clinical symptoms or impaired pulmonary function, the case is classified as ILD [10]. In our study, however, to classify the ILA score on the sole basis of CT images, ILD was defined as abnormalities in > 20% of any lung zone or involvement of more than three zones by ILA without consideration of clinical symptoms and pulmonary function.

The extent of emphysema was determined using the Goddard score [15, 16, 27]. This scoring method evaluates the extent of emphysema on a 5-point scale from 0 to 4 point at three different axial levels in the bilateral lungs: levels of the aortic arch, the carina, and the upper end of the diaphragm. The total score ranges from 0 to 24, with a higher score indicating a larger extent of emphysema. The clinical stage of lung cancer was determined using the eighth edition of the Union for International Cancer Control-Tumor Node Metastasis classification [28].

AEIP was defined as the development of new bilateral ground-glass opacities or consolidation that could not fully be explained by infection, heart failure, volume overload [29], or the spread of lung cancer.

### Data collection from medical charts

Information at the time of lung cancer diagnosis was collected from medical charts; these data included age, sex, smoking index, body mass index (BMI), presence of heart disease, serum creatinine level, serum alanine transaminase level, history of other neoplasm, date of pathological diagnosis of lung cancer, pathological type of lung cancer, recognition as associated with interstitial pneumonia. Information after diagnosis of lung cancer was also obtained from medical charts; these included treatments of lung cancer such as surgery, radiological treatment, chemotherapy; treatment of acute exacerbation of interstitial pneumonia, the use of antifibrotic agents in patients of ILD; date of death, and date of AEIP development. Causes of death were classified into three categories: lung cancer, interstitial pneumonia, and other causes. Death due to AEIP and/or ILD was considered as death due to interstitial pneumonia in the present study.

### Statistical analysis

Background data for the patients are presented as frequency (%) for categorical variables and median (interquartile range) for continuous variables. We evaluated the differences in treatment across stages of non-small cell lung cancer between patients with ILA and ILD using Fisher’s exact test.

Kaplan–Meier curves were generated for all-cause mortality based on each ILA score. Patients who dropped out were considered censored cases on the day of the last visit. Patients who were alive as of December 31, 2020, were also considered censored cases. A log-rank trend test was performed to determine if survival differed according to the ILA score. The Kaplan–Meier curves were also adjusted for the Goddard score, age, year of lung cancer diagnosis, clinical stage of lung cancer, BMI, presence of heart disease, level of serum creatinine, level of serum alanine transaminase, surgery, radiological treatment, and chemotherapy using the inverse probability weighting method [30].

The hazard ratio (HR) for all-cause mortality was calculated using a Cox proportional hazard model with the ILA and Goddard scores as main explanatory variables. The interaction between the ILA score and the Goddard score was also examined. To aid interpretation of the interaction, a histogram of the Goddard score and a figure of estimated HRs for all-cause mortality based on the ILA and Goddard scores were depicted. In addition, the covariates adjusted in the Kaplan–Meier curves were adjusted in the multivariable Cox regression model to avoid theoretical confounding. The missing data consisted of 51 cases of BMI, and 4 of creatinine and alanine transaminase.

In survival analysis, competing risk analysis should be performed when we are interested in more than one outcome [31]. Death due to lung cancer and causes other than interstitial pneumonia was considered a competing risk event in analyses of the development of AEIP. Thus, patients who died of lung cancer and causes other than interstitial pneumonia were treated as censored cases. Cause-specific HRs for the development of AEIP were calculated using the ILA and Goddard scores as main explanatory variables. The interaction between the ILA score and the Goddard score was also examined. The cumulative incidence function for the development of AEIP based on the ILA score was depicted, and the difference in the cumulative incidence function between the different ILA scores was determined using the Gray test.

The same competing analyses with cause-specific hazards and cumulative incidence functions were performed with death due to interstitial pneumonia as a dependent variable. The cumulative incidence functions for death due to lung cancer and causes other than interstitial pneumonia were also depicted for comparison.

All statistical analyses were performed using STATA version 15 (StataCorp LLC, College Station, Texas) and R version 4.2.1 (R Core Team 2022). A two-sided *P* value < 0.05 was considered statistically significant.

## Results

### Prevalence of pulmonary fibrosis and patient background characteristics

**Fig. 1:**
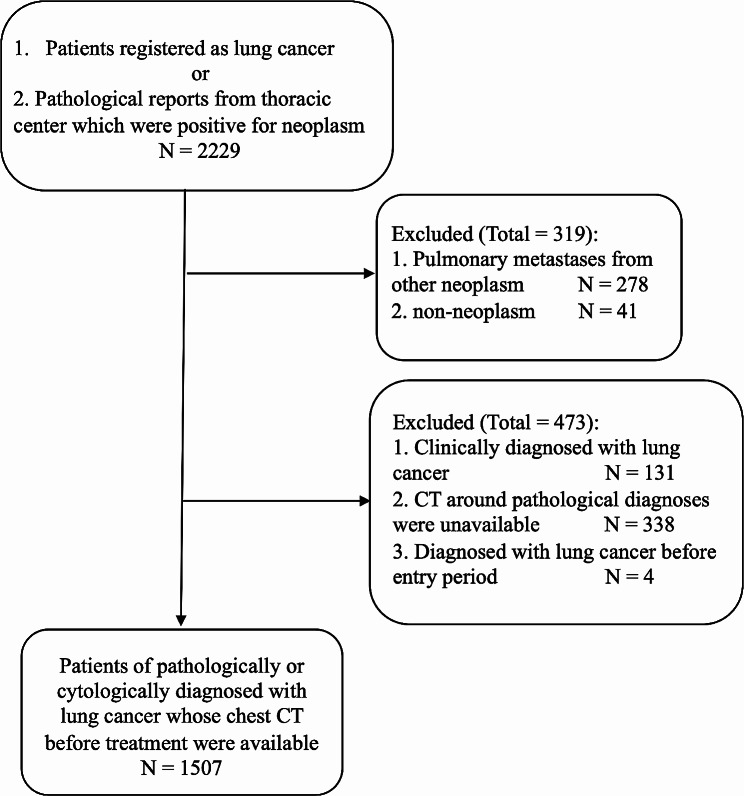
Flowchart describing patient selection CT: computed tomography

We screened 2,229 patients as potential candidates for the present study. After the selection process, depicted in Figs. 1, and 1,507 patients with lung cancer were included. Among these patients, 1,033 (68.5%) exhibited no ILA, 160 (10.6%) had equivocal ILA, 174 (11.5%) had ILA, and 140 (9.3%) had ILD. In total, 474 patients (31.5%) exhibited interstitial pneumonia and 638 patients (42.3%) showed emphysema. While 96.6% of CT images used in this study were reconstructed using a high-resolution algorithm, the median slice thickness was 1.25 mm (interquartile range, 1.00–2.50). Around the time of diagnosis of lung cancer, 5 (3.1%) of equivocal ILA, 61 (35.1%) of ILA, and 130 (92.9%) of ILD were recognized as patients with interstitial pneumonia. Table 1 presents the background characteristics of the patients according to the ILA scores. Male patients, older patients, and patients with squamous cell carcinoma, small cell carcinoma, advanced clinical stage, and a higher smoking index were more likely to have higher ILA scores. Of 140 ILD patients, 13 used antifibrotic medication. Comparison of treatment for non-small cell lung cancer based on stage between ILA and ILD is shown in Table 2. Statistically significant differences were observed in two cases: chemotherapy in stage 1 was more common in the ILD group, while radiation therapy in stage 3 was more common in the ILA group.

**Table 1:** Background characteristics of patients with different interstitial lung abnormality scores

	No ILA (*N* = 1033)	Equivocal ILA (*N* = 160)	ILA (*N* = 174)	ILD (*N* = 140)
Male	546 (52.9)	126 (78.8)	138 (79.3)	120 (85.7)
Age (years)	68 (59, 76)	72 (67, 80)	76 (70, 81)	75 (69, 80)
Pathology				
Adenocarcinoma	827 (80.1)	108 (68.0)	87 (50.0)	58 (41.0)
Squamous cell carcinoma	100 (9.7)	28 (18.0)	57 (33.0)	48 (34.0)
Small cell carcinoma	67 (6.5)	19 (12.0)	21 (12.0)	26 (19.0)
Others	39 (3.8)	5 (3.1)	9 (5.2)	8 (5.7)
Clinical stage				
1	566 (55.0)	90 (56.0)	66 (38.0)	39 (28.0)
2	82 (7.9)	11 (6.9)	23 (13.0)	12 (8.6)
3	122 (12.0)	26 (16.0)	36 (21.0)	39 (28.0)
4	263 (25.0)	33 (21.0)	49 (28.0)	50 (36.0)
Smoking index (pack-years)	12 (0, 44)	40 (14, 56)	46 (25, 76)	50 (40, 71)
Body mass index (kg/m^2^)	21.8 (19.6, 24.1)	23.1 (21.0, 25.4)	22.0 (20.1, 24.6)	22.8 (20.5, 24.7)
Heart disease	130 (12.6)	35 (21.9)	41 (23.6)	37 (26.4)
Creatinine (mg/dL)	0.72 (0.60, 0.84)	0.79 (0.68, 0.94)	0.82 (0.67, 0.99)	0.79 (0.67, 0.92)
Alanine transaminase (international unit/L)	18 (14, 24)	17 (14, 25)	16.5 (13, 23)	17 (13, 27)
History of other neoplasm	205 (19.8)	40 (25.0)	47 (27.0)	23 (16.4)
Surgery	576 (55.8)	91 (56.9)	74 (42.5)	43 (30.7)
Radiation therapy	174 (16.8)	40 (25.0)	29 (16.7)	9 (6.4)
Chemotherapy	385 (37.3)	58 (36.3)	59 (33.0)	59 (42.1)

Data are presented as number (%) or median (interquartile range)

ILA: interstitial lung abnormality; ILD: interstitial lung disease

**Table 2:** Comparison of treatment for non-small cell lung cancer based on stage between interstitial lung abnormality and interstitial lung disease

	ILA (*N* = 153)	ILD (*N* = 114)	*P* value
Stage 1	*N* = 66	*N* = 37	
Surgery	53 (80.3)	30 (81.1)	1.000
Radiotherapy	13 (19.7)	3 (8.1)	0.160
Chemotherapy	10 (15.2)	12 (32.4)	0.048
Best supportive care	3 (4.5)	3 (8.1)	0.664
Stage 2	*N* = 23	*N* = 11	
Surgery	14 (60.9)	7 (63.6)	1.000
Radiotherapy	4 (17.4)	0 (0.0)	0.280
Chemotherapy	6 (26.1)	2 (18.2)	1.000
Best supportive care	4 (17.4)	4 (36.4)	0.388
Stage 3	*N* = 32	*N* = 30	
Surgery	5 (15.6)	4 (13.3)	1.000
Radiotherapy	8 (25.0)	0 (0.0)	0.005
Chemotherapy	14 (43.8)	14 (46.7)	1.000
Best supportive care	12 (37.5)	14 (46.7)	0.607
Stage 4	*N* = 32	*N* = 36	
Surgery	2 (6.2)	0 (0.0)	0.218
Radiotherapy	0 (0.0)	3 (8.3)	0.214
Chemotherapy	15 (46.9)	12 (33.3)	0.323
Best supportive care	15 (46.9)	22 (61.1)	0.330

Data are presented as number (%). Patients may receive multiple types of treatment

Best supportive care includes cases where patients choose only best supportive care from the time of lung cancer diagnosis

ILA: interstitial lung abnormality; ILD: interstitial lung disease

### All-cause mortality

**Fig. 2:**
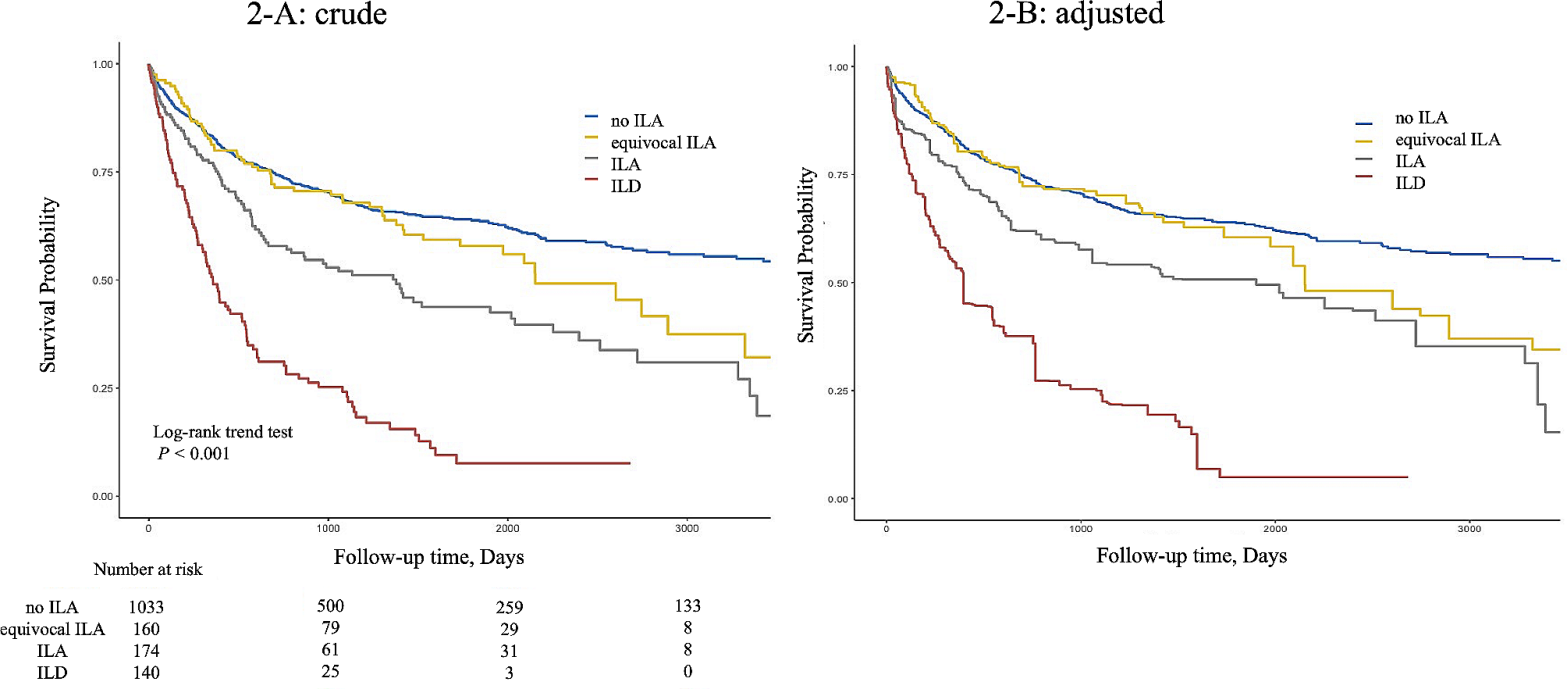
Crude (**A**) and adjusted (**B**) Kaplan–Meier curves for all-cause mortality based on the interstitial lung abnormality score The curve in B is made after adjustment for the following variables: Goddard score, age, clinical stage of lung cancer, year of diagnosis of lung cancer, body mass index, heart disease, serum creatinine level, serum alanine transaminase level, history of other neoplasms, surgery, radiational therapy, and chemotherapy ILA: interstitial lung abnormality; ILD: interstitial lung disease

Figure 2A shows the Kaplan–Meier curve for all-cause mortality according to each ILA score. The median follow-up duration was 817 days. The log-rank trend test indicated that the survival probability significantly improved with a decrease in the ILA score: no ILA > equivocal ILA > ILA > ILD (*P* < 0.001). The Kaplan–Meier curve with adjustment for covariates is illustrated in Fig. 2B.

**Fig. 3:**
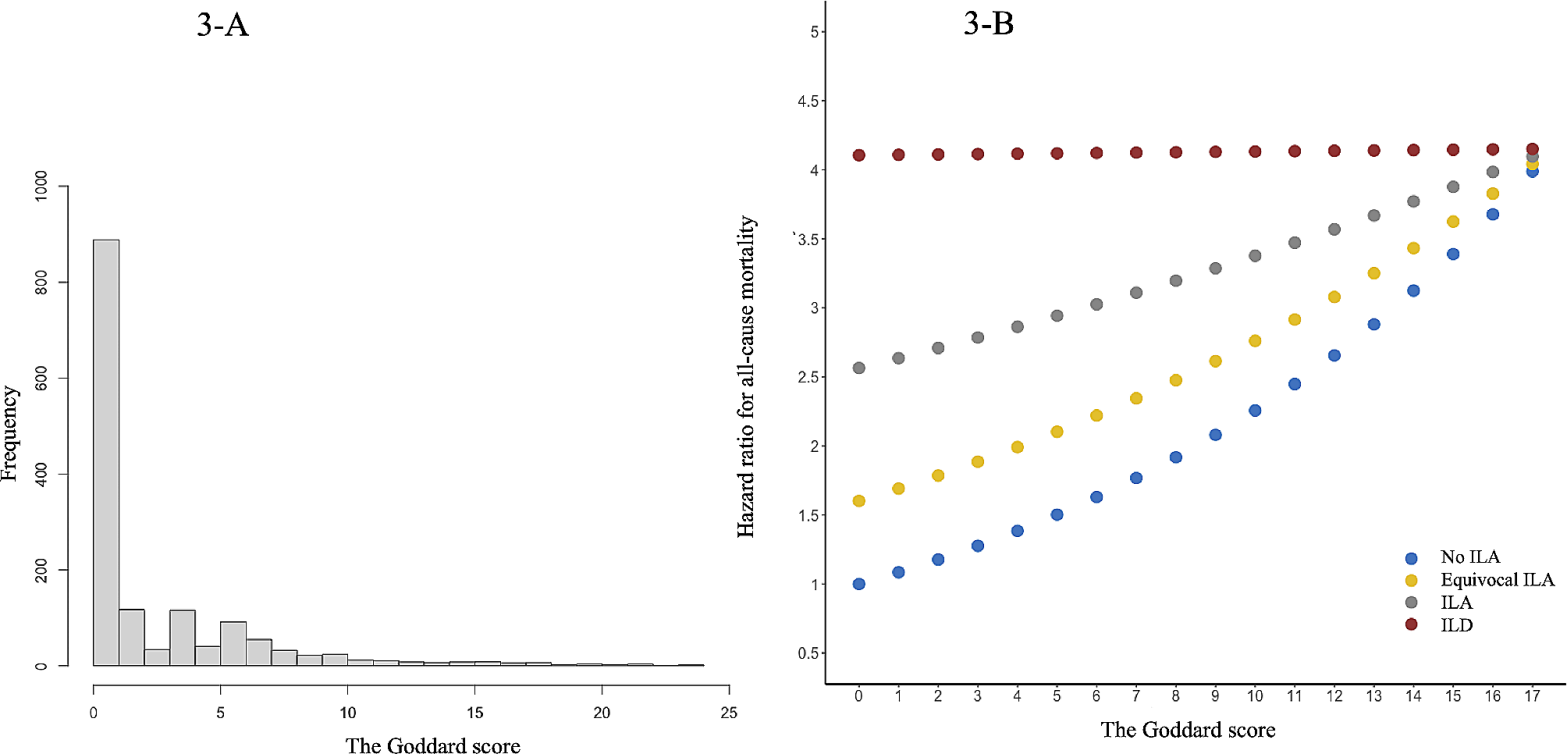
Histogram for the Goddard score and hazard ratio for all-cause mortality estimated by the interstitial lung abnormality and Goddard scores ILA: interstitial lung abnormality; ILD: interstitial lung disease

Crude Cox regression analysis indicated that the ILA and Goddard scores were associated with increased HRs for all-cause mortality as follows: no ILA (HR, 1.00: reference), equivocal ILA (HR, 1.60; 95% confidence interval [CI], 1.45–1.77; *P* < 0.001), ILA (HR, 2.56; 95% CI, 2.10–3.13; *P* < 0.001), ILD (HR, 4.11; 95% CI, 3.04–5.54; *P* < 0.001), and Goddard score (HR, 1.08; 95% CI, 1.06–1.11; *P* < 0.001; Table 2). Moreover, there was a significant interaction between the ILA score and the Goddard score (*P* < 0.001). Figure 3 shows the distribution of the Goddard score (A) and the comprehensive impact of interstitial pneumonia and emphysema with the crude interaction between the ILA score and the Goddard score (B); the impact of the Goddard score on all-cause mortality decreased as the ILA score increased. After adjustment for multiple covariates, the ILA and Goddard scores remained associated with increased HRs for all-cause mortality (Table 3).

**Table 3:** Hazard ratios for all-cause mortality according to the interstitial lung abnormality and Goddard scores

Variables	Crude Cox plus interaction			Adjusted Cox plus interaction		
	HR	95% CI	*P* value	HR	95% CI	*P* value
No ILA	1.00	Reference		1.00	Reference	
Equivocal ILA	1.60	1.45–1.77	< 0.001	1.31	1.18–1.46	< 0.001
ILA	2.56	2.10–3.13	< 0.001	1.71	1.39–2.12	< 0.001
ILD	4.11	3.04–5.54	< 0.001	2.24	1.63–3.09	< 0.001
Goddard score	1.08	1.06–1.11	< 0.001	1.03	1.01–1.06	0.010
No ILA * GS ^a^	1.00	Reference		1.00	Reference	
Equivocal ILA * GS ^a^	0.97	0.96–0.99	< 0.001	0.98	0.96–0.99	0.005
ILA * GS ^a^	0.95	0.92–0.98	< 0.001	0.96	0.93–0.99	0.005
ILD * GS ^a^	0.92	0.88–0.96	< 0.001	0.94	0.89–0.98	0.005
Age (per 1 year)	1.04	1.04–1.05	< 0.001	1.02	1.01–1.03	< 0.001
Year of diagnosis	0.94	0.92–0.96	< 0.001	0.97	0.95–1.00	0.024
Clinical stage	2.58	2.41–2.77	< 0.001	2.04	1.81–2.30	< 0.001
Body mass index	0.93	0.91–0.95	< 0.001	0.98	0.95–1.00	0.026
Heart disease	1.84	1.52–2.23	< 0.001	1.30	1.05–1.61	0.014
Creatinine	1.07	0.98–1.17	0.136	1.11	0.99–1.25	0.081
Alanine transaminase	1.01	1.00–1.01	< 0.001	1.00	1.00–1.01	< 0.001
History of other neoplasm	1.01	0.83–1.22	0.947	1.19	0.96–1.47	0.111
Adenocarcinoma	1.00	Reference		1.00	Reference	
Squamous cell carcinoma	2.48	2.02–3.06	< 0.001	1.46	1.15–1.85	0.002
Small cell carcinoma	4.35	3.47–5.44	< 0.001	1.74	1.34–2.25	< 0.001
Others	2.00	1.37–2.93	< 0.001	1.56	1.03–2.36	0.038
Surgery	0.09	0.07–0.11	< 0.001	0.26	0.19–0.36	< 0.001
Radiation therapy	1.33	1.10–1.61	0.004	0.63	0.49–0.81	< 0.001
Chemotherapy	2.64	2.09–2.90	< 0.001	0.61	0.49–0.77	< 0.001

The interaction between the interstitial lung abnormality score and the Goddard score for all-cause mortality is also significant

HR: hazard ratio; CI: confidence interval; ILA: interstitial lung abnormality; ILD: interstitial lung disease; GS: Goddard score

^a^ Interaction term

### AEIP

**Fig. 4:**
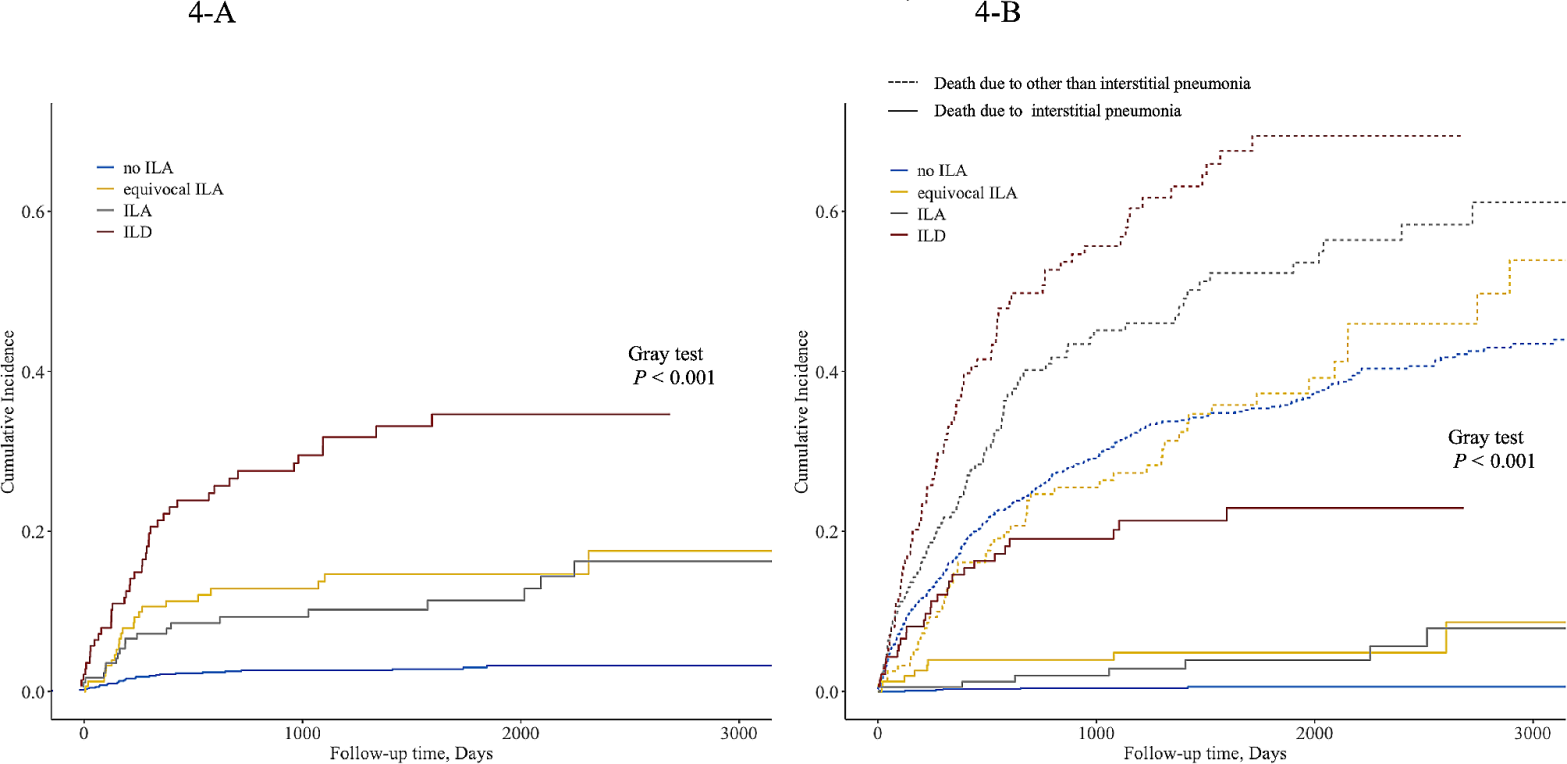
Cumulative incidence curves for the development of acute exacerbation of interstitial pneumonia and death due to interstitial pneumonia (**A**) development of acute exacerbation of interstitial pneumonia. (**B**) death due to interstitial pneumonia ILA: interstitial lung abnormality; ILD: interstitial lung disease

Of 111 acute exacerbations, 47 resulted in mortality due to the progression of interstitial pneumonia. In terms of treatment of AEIP, glucocorticoids were administered in 100 cases. The ILA and Goddard scores were both significantly associated with increased cause-specific HRs for two outcomes: development of AEIP and death due to interstitial pneumonia (Table 4). We also demonstrated a significant interaction between the two scores for both outcomes (*P* < 0.001, *P* = 0.002). The cumulative incidence function indicated that patients with equivocal ILA had a higher incidence of both outcomes than did patients with no ILA (Fig. 4A, B). Furthermore, patients with ILD had the highest cumulative incidence of both outcomes (Fig. 4A, B). Frequency of AEIP based on both stage and treatment of lung cancer is shown in Table 5.

**Table 4:** Cause-specific hazard ratios for the development of acute exacerbation of interstitial pneumonia and death from interstitial pneumonia according to the interstitial lung abnormality and Goddard scores

	Cause-specific hazard ratio for development for acute exacerbation of interstitial pneumonia			Cause-specific hazard ratio for death due to interstitial pneumonia		
	HR	95% CI	*P* value	HR	95% CI	*P* value
No ILA	1.00	Reference		1.00	Reference	
Equivocal ILA	2.99	2.42–3.69	< 0.001	5.08	3.45–7.46	< 0.001
ILA	8.91	5.84–13.60	< 0.001	25.76	11.93–55.65	< 0.001
ILD	26.61	14.11–50.16	< 0.001	130.78	41.20–415.13	< 0.001
Goddard score	1.12	1.06–1.19	< 0.001	1.18	1.07–1.31	0.002
No ILA * GS ^a^	1.00	Reference		1.00	Reference	
Equivocal ILA * GS ^a^	0.94	0.92–0.97	< 0.001	0.93	0.88–0.97	0.002
ILA * GS ^a^	0.89	0.84–0.95	< 0.001	0.86	0.78–0.95	0.002
ILD * GS ^a^	0.84	0.77–0.92	< 0.001	0.80	0.69–0.92	0.002

The interaction between the interstitial lung abnormality score and the Goddard score is also significant

HR: hazard ratio; CI: confidence interval; ILA: interstitial lung abnormality; ILD: interstitial lung disease; GS: Goddard score

^a^ interaction term

**Table 5:** Frequency of acute exacerbation of interstitial pneumonia based on both stage and treatment of lung cancer

Stage of lung cancer	Acute exacerbation of interstitial pneumonia
Probable causes of AEIP	
Stage 1 (*N* = 761)	33 (4.3)
Surgery (*N* = 652)	3 (0.5)
Radiotherapy (*N* = 97)	13 (13.4)
Chemotherapy (*N* = 125)	12 (9.6)
Unknown causes	8
Stage 2 (*N* = 128)	5 (3.9)
Surgery (*N* = 79)	0 (0.0)
Radiotherapy (*N* = 30)	2 (6.7)
Chemotherapy (*N* = 62)	0 (0.0)
Unknown causes	3
Stage 3 (*N* = 223)	44 (19.7)
Surgery (*N* = 46)	2 (4.3)
Radiotherapy (*N* = 97)	18 (18.6)
Chemotherapy (*N* = 136)	17 (12.5)
Unknown causes	10
Stage 4 (*N* = 395)	29 (7.3)
Surgery (*N* = 7)	1 (14.3)
Radiotherapy (*N* = 28)	4 (14.3)
Chemotherapy (*N* = 238)	20 (8.4)
Unknown causes	5

Data are presented as number (%) or number. The causes of acute exacerbation of interstitial pneumonia may overlap

AEIP: acute exacerbation of interstitial pneumonia

## Discussion

The present study revealed four main findings. First, 31.5% of patients with lung cancer showed various extents of interstitial pneumonia. Second, HRs of all-cause mortality were increased in the following order: no ILA (score 0), equivocal ILA (score 1), ILA (score 2), and ILD (score 3). Third, cause-specific HRs for the development of AEIP also sequentially increased with an increase in the ILA score. Fourth, HRs of these outcomes increased with an increase in the Goddard score; this allowed evaluation of the comprehensive impact of interstitial pneumonia and emphysema on patients with lung cancer using the ILA and Goddard scores, with a significant interaction between the two scores.

Our study demonstrated that a significant proportion (31.5%) of patients diagnosed with lung cancer exhibited varying degrees of interstitial pneumonia. The prevalence of IPF in patients with lung cancer has been reported to range from 7.5 to 16.8% in previous studies [3–5]. By encompassing trivial, mild, and diverse forms of interstitial pneumonia, up to 31.5% of lung cancer cases may be considered to have interstitial pneumonia. Earlier investigations also reported that 40.7% of lung cancer patients had equivocal ILA or ILA [11]. Consequently, the presence of various degrees of interstitial pneumonia among lung cancer patients could no longer be regarded as a minor occurrence, which is not unexpected because age and tobacco are common risk factors for ILA and lung cancer [10, 32]. Notably, in our study, the recognition of trivial or mild interstitial pneumonia states has not been as comprehensive as that of advanced state. However, as discussed further, even trivial interstitial pneumonia may result in poorer prognosis and increased incidence of AEIP. Therefore, we emphasize the need for heightened awareness of interstitial pneumonia, including even trivial manifestations.

Our study showed that HRs of all-cause mortality increased consecutively with an increase in the ILA score. These results are consistent with those of previous studies reporting that ILA and IPF are associated with higher mortality rates in patients with lung cancer [3–5, 11–13]. A case–control study demonstrated that patients with lung cancer complicated by IPF had a poorer prognosis than did those with ILA [13]. Our study further revealed that even equivocal ILA worsened the prognosis in lung cancer patients and that the extent of interstitial pneumonia influenced their prognosis. Previous studies recruited patients with a specific clinical stage of specific lung cancers [3–5, 11–13]. Because our study included all clinical stages of both small cell lung cancer and non-small cell lung cancer, our results could help in understanding the gross impact of interstitial pneumonia in patients with lung cancer.

In the present study, cause-specific HRs for AEIP were observed to increase sequentially with an increase in the ILA score. These findings are consistent with those shown in prior research that reported increased odds ratios for AEIP in patients with lung cancer complicated by either IPF or ILA [6, 7, 33, 34]. Our study also revealed that even equivocal ILA was associated with increased HRs for the development of AEIP. In previous studies, analyses of AEIP in patients with lung cancer were reported with frequencies or odds ratios based on logistic regression analyses [6, 7, 33, 34]. However, studies of lung cancer generally censor their data. Therefore, survival analyses incorporating censored data could present less biased results. Furthermore, because death due to lung cancer could be a major competing risk event for AEIP, competing risk analysis is warranted for the evaluation of AEIP in patients with lung cancer. We believe that our study presents results based on less biased analyses.

We could evaluate the comprehensive impact of interstitial pneumonia and emphysema on mortality in patients with lung cancer on the basis of the ILA and Goddard scores and the interaction between the two scores. With regard to all-cause mortality, HRs increased with an increase in the two scores. Conversely, the impact of the Goddard score for all-cause mortality decreased with an increase in the ILA score. Prior studies have focused on the individual influence of ILD, ILA, and emphysema in patients with lung cancer [3–5, 11–13, 15–17]. Nevertheless, ILD and ILA are often associated with emphysema [14], and the impact of concomitant emphysema has not been explored. Moreover, studies of CPFE in patients with lung cancer treated interstitial pneumonia, emphysema, and CPFE as separate diseases [20–25]. Therefore, information regarding isolated interstitial pneumonia was not included as part of data for CPFE, and information regarding interstitial pneumonia in CPFE was not included as part of data for isolated interstitial pneumonia; this may have resulted in biased and inaccurate estimations. The same limitation existed for isolated emphysema and CPFE. Moreover, for diagnosis of CPFE, the presence of interstitial pneumonia and emphysema were dichotomously assessed without incorporation of the extent of interstitial pneumonia and emphysema into the analyses [20–25]. However, by combining the ILA and Goddard scores and their interaction, we can overcome these limitations and comprehensively evaluate the influence of interstitial pneumonia and emphysema on patients with lung cancer.

Our findings have important clinical implications. In patients with lung cancer complicated by interstitial pneumonia, there is inadequate evidence of improved prognosis with any treatment [35], and any intervention can trigger AEIP [6, 7, 33, 34]. Using our results, physicians can estimate individual HRs for all-cause mortality and AEIP based on the ILA and Goddard scores before proceeding with a treatment for lung cancer. Even in patients with equivocal ILA, physicians should carefully consider the risks of AEIP.

The present study had some limitations. First, it was a retrospective cohort study conducted in a single institution, which raises the possibility of selection bias. Further studies are warranted to validate and generalize our findings. Second, we could not assess the performance status, which is an important factor for the prognosis of patients with lung cancer. Third, data regarding pulmonary function tests and the pathology of interstitial pneumonia were lacking, despite their potential implications for the prognosis. Patients with stage 3 or 4 lung cancer often exhibit a poor general condition, which could make it difficult to conduct pulmonary function tests or obtain pathological specimens of interstitial pneumonia without missing cases.

## Conclusions

Our results suggest that approximately one-third of patients with lung cancer have interstitial pneumonia when incorporating trivial and mild cases. Because interstitial pneumonia and emphysema, ranging from trivial to severe, significantly impact all-cause mortality and the development of AEIP in patients with lung cancer, we should recognize trivial and mild forms of these pulmonary abnormalities in addition to their advanced states. Moreover, the influence of CPFE could be quantified by considering the collective extent of interstitial pneumonia and emphysema and the interaction between these conditions.

## Data Availability

The datasets used and/or analyzed during the current study are available from the corresponding author on reasonable request.

## Declarations

### Clinical trial number

As our research is not a prospective study, we do not have a Clinical Trial Number.

## Abbreviations

AEIP: Acute exacerbation of interstitial pneumonia
BMI: Body mass index
CI: Confidence interval
CPFE: Combined pulmonary fibrosis and emphysema
CT: Computed tomography
HR: Hazard ratio
ILA: Interstitial lung abnormality
ILD: Interstitial lung disease
IPF: Idiopathic pulmonary fibrosis
